# Global and Local Manipulation of DNA Repair Mechanisms to Alter Site-Specific Gene Editing Outcomes in Hematopoietic Stem Cells

**DOI:** 10.3389/fgeed.2020.601541

**Published:** 2020-12-10

**Authors:** Elizabeth K. Benitez, Anastasia Lomova Kaufman, Lilibeth Cervantes, Danielle N. Clark, Paul G. Ayoub, Shantha Senadheera, Kyle Osborne, Julie M. Sanchez, Ralph Valentine Crisostomo, Xiaoyan Wang, Nina Reuven, Yosef Shaul, Roger P. Hollis, Zulema Romero, Donald B. Kohn

**Affiliations:** ^1^Department of Microbiology, Immunology & Molecular Genetics, David Geffen School of Medicine, University of California, Los Angeles, Los Angeles, CA, United States; ^2^Department of General Internal Medicine and Health Services Research, University of California, Los Angeles, Los Angeles, CA, United States; ^3^Department of Molecular Genetics, Weizmann Institute of Science, Rehovot, Israel

**Keywords:** gene editing, hematopoietic stem cells, DNA repair, Cas9, HDR, sickle cell disease

## Abstract

Monogenic disorders of the blood system have the potential to be treated by autologous stem cell transplantation of *ex vivo* genetically modified hematopoietic stem and progenitor cells (HSPCs). The sgRNA/Cas9 system allows for precise modification of the genome at single nucleotide resolution. However, the system is reliant on endogenous cellular DNA repair mechanisms to mend a Cas9-induced double stranded break (DSB), either by the non-homologous end joining (NHEJ) pathway or by the cell-cycle regulated homology-directed repair (HDR) pathway. Here, we describe a panel of ectopically expressed DNA repair factors and Cas9 variants assessed for their ability to promote gene correction by HDR or inhibit gene disruption by NHEJ at the *HBB* locus. Although transient global overexpression of DNA repair factors did not improve the frequency of gene correction in primary HSPCs, localization of factors to the DSB by fusion to the Cas9 protein did alter repair outcomes toward microhomology-mediated end joining (MMEJ) repair, an HDR event. This strategy may be useful when predictable gene editing outcomes are imperative for therapeutic success.

## Introduction

Inherited disorders of the hematopoietic system, such as primary immune deficiencies and hemoglobinopathies, have been historically treated by allogeneic hematopoietic stem cell transplantation (HSCT) of healthy HLA-matched donor cells (Griffith et al., 2008). The self-renewing hematopoietic stem and progenitor cells (HSPCs) engraft and repopulate the bone-marrow niche of a conditioned recipient, providing a steady supply of healthy blood cells. However, allogeneic HSCT does not come without risks; recipients may suffer graft rejection, graft-vs.-host disease, or complications due to immunosuppression (Dvorak and Cowan, 2008; Pai et al., 2014). Gene modification of autologous HSPCs for transplantation can circumvent these risks. Current approaches utilize site-specific endonucleases to facilitate precise gene editing, with the Cas family of RNA-guided nucleases emerging as the most promising for therapeutic gene editing. Of these, the *Streptococcus pyogenes* Cas9 (SpCas9) enzyme is recognized for its ease of use and production, and remarkable ability to hone in on a 20 base pair sequence among the ~3 billion base pairs in the human genome to create a directed double-stranded break (DSB; Doudna and Charpentier, 2014). Once the DSB is introduced, endogenous cell repair mechanisms are employed to mend the lesion.

Two main pathways compete to repair the break: non-homologous end joining (NHEJ), an imprecise repair pathway that can result in insertions and deletions (indels), or accurate homology-directed repair (HDR), which uses a donor template to seamlessly repair the break in S/G2 phases of cell cycle (Sartori et al., 2007; Branzei and Foiani, 2008; Heyer et al., 2010; Pietras et al., 2011; Symington and Gautier, 2011; Fradet-Turcotte et al., 2013; Jasin and Rothstein, 2013; Panier and Boulton, 2014; Polato et al., 2014; Anand et al., 2016; Cuella-Martin et al., 2016; Jasin and Haber, 2016; Symington, 2016; Lomova, 2019; Romero et al., 2019; Ceppi et al., 2020). Additionally, recent work suggests that microhomology-mediated end joining (MMEJ), an HDR event that results in deletions, is also a notable repair pathway in many cell types (McVey and Lee, 2008; Huertas, 2010; Iyer et al., 2019; Wu et al., 2019; Yeh et al., 2019). To accurately repair the DSB and introduce specific sequence changes to the gene, a DNA donor template designed with single nucleotide polymorphisms (SNPs) and flanked by homology arms can be incorporated into the genome via HDR. The activity of the repair pathways is not equivalent; NHEJ is more prevalent than HDR in mammalian cells (Chiruvella et al., 2013, Yeh et al., 2019). For certain diseases, where a knockout of a gene can result in therapeutic benefit, repair by the NHEJ pathway is favorable (Holt et al., 2010; Bauer et al., 2013; Bjurström et al., 2016; Chang et al., 2017). However, for site-specific gene correction of sickle cell disease (SCD), where disruption of the target *HBB* gene can result in a different or more severe disease phenotype, correction via HDR pathway is critical.

In the last several years, there have been many efforts to control DNA repair outcomes for genome editing by either globally inhibiting or activating DNA repair factors (DNA RFs; Yeh et al., 2019). Numerous studies have shown improvements in HDR or inhibition of NHEJ repair through overexpression of factors that promote or restrict these pathways, respectively (Orthwein et al., 2015; Canny et al., 2018; Supplementary Figures 1A–C). However, the effects of these manipulations on primary human HSPCs have not been previously reported. Local manipulation of DNA repair factors to control editing outcomes may prove to be a superior strategy over global manipulation of DNA repair. Cell cycle control of HDR to specific HDR-permissive states protects against loss of heterozygosity, while the NHEJ pathway is primarily in place as a protective mechanism against the estimated 10–50 DNA lesions that occur in a cell per day through natural causes (Ellis et al., 1995; Vilenchik and Knudson, 2003; Yeh et al., 2019). Localization of DNA RFs to a Cas9-induced DSB may reduce the risks associated with global manipulation of DNA repair (Jayavaradhan et al., 2019). Furthermore, tethering DNA RFs to Cas9 may ensure that the factors are present and active as soon as a Cas9-induced DSB occurs, thus controlling the fate of repair outcomes. Recent efforts of local manipulation of DNA repair factors have reported successes in cell lines. Fusion of the “HDR enhancer element of CtIP” to Cas9 or Cas9-hGeminin (Cas9-hCtIP and Cas9-hGem-hCtIP, respectively) effectively increased HDR (Charpentier et al., 2018). Tethering of a dominant negative form of 53BP1 (DN1S) to Cas9 was able to inhibit NHEJ while maintaining levels of HDR (Jayavaradhan et al., 2019). To date, the only Cas9 fusion variant shown to improve the HDR/NHEJ ratio in primary HSPCs is Cas9-hGem (Gutschner et al., 2016; Lomova et al., 2019).

In this study, we investigated the cellular elements that govern the DNA repair pathway choice and how they can be exploited to shift the balance from NHEJ toward HDR while targeting the SCD causative mutation in *HBB*. We evaluated whether global overexpression of a series of DNA RFs can improve gene editing levels in a K562 cell line and primary CD34^+^ human HSPCs. Interestingly, we observed no consistent improvement in HDR by over-expression of any of the DNA RF we examined, although there was non-specific improvement in HDR in K562 cells by the addition of plasmid DNA. In a parallel approach, we tested a panel of Cas9 variants fused to DNA RFs for their ability to promote HDR or inhibit NHEJ specifically at the DSB. Variants containing a fragment of the human Geminin (hGem) protein consistently reduced the frequency of NHEJ alleles compared to Cas9, while the levels of HDR remained similar. We observed an increase in MMEJ signature when HSPCs were edited with Cas9-hCtIP variants, suggesting that the CtIP fusion is biologically active but does not promote gene correction by canonical HDR.

## Results

### Evaluating the Effects of DNA RF Overexpression on Gene Editing Levels in K562 Cells

In human cell lines, it has been shown that constitutively active phosphomimetic forms of CtIP (CtIP T847E, denoted as CtIP^E^; CtIP S249D T847E, denoted as CtIP^DE^), can promote end resection in G1 phase of the cell cycle and recruit BRCA1 irrespective of cell cycle stage (Huertas and Jackason, 2009; Orthwein et al., 2015). Furthermore, modifying PALB2 with mutations in the BRCA1 binding pocket (PALB2^KR^) results in cell cycle-independent interaction with BRCA1; when coupled with activation of DSB end resection, HDR can occur in G1 (Orthwein et al., 2015). Inhibition of NHEJ factors can be beneficial by either limiting undesired indels or skewing repair toward HDR. Inhibitor of 53BP1 (i53) targets the ubiquitin-dependent recruitment (UDR) domain of 53BP1, preventing its recruitment to DSB and stimulating HDR (Canny et al., 2018; Supplementary Figure 1B). A truncated fragment of 53BP1 containing an identical tandem Tudor domain competitively antagonize the protein in a dominant negative fashion (dn53BP1). Coupled with ectopic expression of RAD52, dn53BP1 has been shown to improve HDR through the single strand template repair (SSTR) pathway (Paulsen et al., 2017).

**Figure 1:**
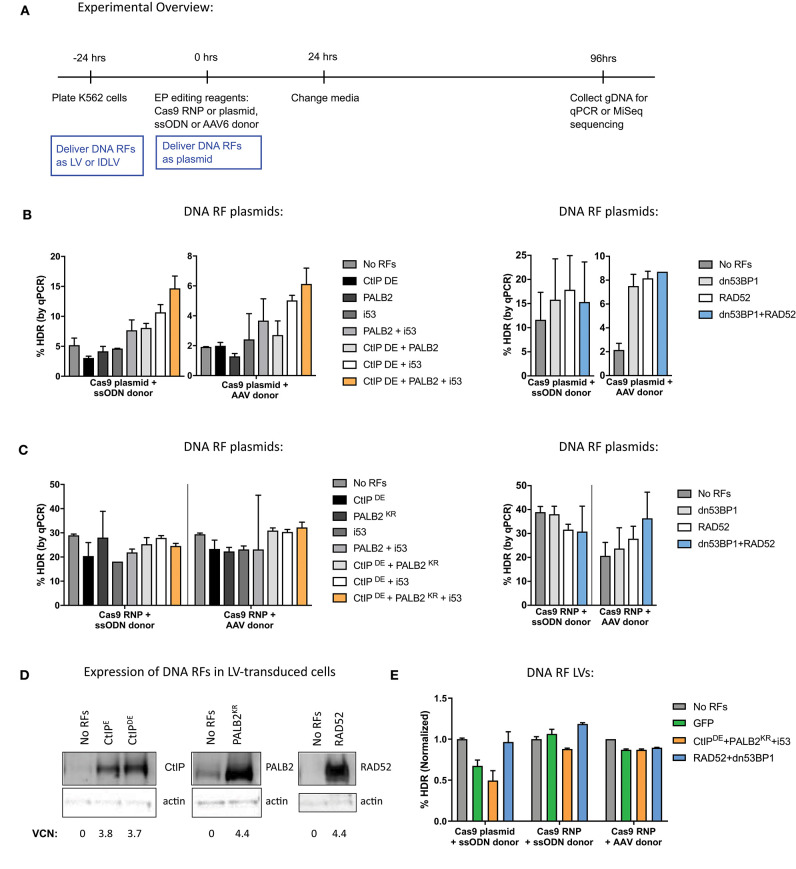
The method for delivery of DNA repair factors (RFs) for global overexpression in K562 differentially affects gene editing outcomes. **(A)** Experimental overview of K562 cell transduction and electroporation for delivery of DNA RFs and editing reagents. DNA RFs delivered as plasmid were co-electroporated with editing reagents, while delivery of DNA RFs as LVs occurred 24 h prior to electroporation. **(B,C)** Cas9 nuclease was delivered either as plasmid (1 μg) or RNP (100 pmol Cas9 protein + 4.5 μg of IVT sgRNA); donor template was delivered either as ssODN (3 μM) or AAV6 (MOI 2e4). DNA RFs were delivered as a plasmid. HDR levels were measured by qPCR. *n* = 2 biological replicates for CtIP^DE^ + PALB2^KR^ + i53 experiments, *n* = 6 biological replicates for RAD52 + dn53BP1 experiments. Error bars, mean ± SD. **(D)** K562 cells were transduced with LVs expressing the indicated DNA RFs. Western Blot was performed on day 10 post-transduction. Vector copy number (VCN) was determined by droplet digital PCR (ddPCR). **(E)** K562 cells were transduced with DNA RF LVs and electroporated with editing reagents. *n* = 2 biological replicates. Data are normalized to “No RFs” conditions for each set of experiments. Error bars, mean ± SD.

To evaluate the effects of DNA RFs on HDR and NHEJ levels, factors were overexpressed from MND-LTR-U3-driven expression plasmids by co-electroporation with gene editing reagents into K562 cells, erythroleukemia cell line that is commonly used as a proxy for HSPCs (Figure 1A; see Table 1 for a list of DNA RFs tested). Cas9 and the single guide RNA (sgRNA) to *HBB* were delivered either as expression plasmids or ribonucleoprotein (RNP), and donor template was delivered either as a single-stranded oligodeoxynucleotide (ssODN), or an adeno-associated virus 6 (AAV6; DeWitt et al., 2016; Lomova et al., 2019; Romero et al., 2019). The percentage of gene correction was measured by qPCR (Hoban et al., 2015).

**Table 1:** Panel of DNA repair factors assessed in this study.

**DNA RFs**	**Description**
CtIP^E^/CtIP^DE^	T847E mutant acts as a CDK- mediated phosphomimetic.; S249D mutant increases BRCA2 recruitment to DSB (Orthwein et al., 2015)
PALB2^KR^	KR mutation in the BRCA1 binding pocket allows for PALB2/BRCA1 binding irrespective of cell cycle (Orthwein et al., 2015)
i53	“Inhibitor of 53BP1” is an ubiquitin variant that binds to 53BP1 and prevents its accumulation at a DSB (Canny et al., 2018)
RAD52	Improves SSTR (Paulsen et al., 2017)
dn53BP1	Dominant negative form of 53BP1; inhibits NHEJ (Paulsen et al., 2017)

When Cas9 was delivered as a plasmid, a combination of CtIP^DE^, PALB2^KR^, and i53 improved HDR levels almost 3-fold, compared to “No RFs” control with both ssODN and AAV donors. However, overexpression of the factors individually did not have any significant effects on HDR levels. Similar improvements in HDR levels were observed when RAD52 and dn53BP1 plasmids were co-electroporated with Cas9 plasmid (Figure 1B).

In contrast, when the same DNA RFs were co-delivered as plasmids with Cas9 RNP (Figure 1C), there were no improvements in HDR levels when CtIP^DE^, PALB2^KR^, and i53 were expressed in combination, irrespective of donor template used for repair. No improvements in HDR levels were detected in the context of ssODN donor when RAD52 and dn53BP1 were used in combination. A slight improvement (1.5-fold) in HDR levels in the context of AAV6 donor was observed with RAD52 and dn53BP1. We hypothesized that the reason for not achieving improvements in HDR levels when delivering Cas9 as RNP and DNA RFs as plasmids was due to delayed kinetics of DNA RF transcription and translation from the plasmid, relative to Cas9 RNP, which is already in its active protein form at the time of electroporation into the cells.

To synchronize expression of the DNA RFs during Cas9 RNP editing, cells were transduced with lentiviruses (LVs) expressing DNA RFs, 24 h prior to electroporation of gene editing reagents. To confirm overexpression, K562 cells were transduced with LVs at multiplicity of infection (MOI) of 1, and DNA RF protein expression was assessed by western blot analysis (Figure 1D). The results showed basal expression of CtIP^E^, CtIP^DE^, PALB2, and RAD52 proteins in “No RFs” condition (untransduced samples) and confirmed protein overexpression in LV-transduced samples. Western blots for i53 and dn53BP1 were not performed due to unavailability of selective antibodies for these inhibitors. Analysis of editing outcomes revealed that expression of the DNA RFs from LVs did not have an effect on HDR levels, compared to “No RF” or GFP controls, independently of the way that Cas9 was delivered (plasmid vs. RNP) and the DNA donor template used (ssODN vs. AAV6; Figure 1E).

To test whether the improvements in HDR levels observed in Figure 1B were a true effect of the DNA RFs or merely a result of plasmid co-electroporation, we tested a GFP control plasmid co-electroporated at varying amounts (0.3–10 μg) with Cas9 and sgRNA plasmid delivery. The levels of gene editing were measured by high throughput sequencing (HTS) of the *HBB* target site. Increases in both HDR and NHEJ were detected with the addition of increasing amounts of GFP control plasmid (Supplementary Figure 2A). These data suggest that the increases in HDR levels observed earlier might be an artifact of plasmid co-electroporation and not the biological effect of DNA RFs.

Next, we went on to compare delivery of DNA RFs and GFP control as LV, integrase-defective lentiviral vector (IDLV) or plasmid. K562 cells were transduced with LV and IDLV 24 h prior to electroporation of gene editing reagents at a MOI that resulted in similar GFP expression to GFP plasmid electroporation at 24 h (refer to Figure 1A for timeline). Although not statistically significant, all plasmids (CtIP^DE^ + PALB2^KR^ + i53, RAD52 + dn53BP1, and GFP control) increased both HDR and NHEJ levels ~2-fold, while none of the LVs or IDLVs had an effect on either HDR or NHEJ (Supplementary Figure 2B). Of note, additional transduction timepoints and varying MOIs were tested, but still did not improve HDR levels (data not shown). Together, these data suggest that plasmid co-electroporation induced a response in K562 cells that increased DNA repair levels via both HDR and NHEJ pathways. However, it does not appear that the overexpression of ectopic DNA RFs directly improved HDR levels.

### Evaluating the Effects of DNA RF Overexpression on Gene Editing Levels in Primary Human HSPCs

**Figure 2:**
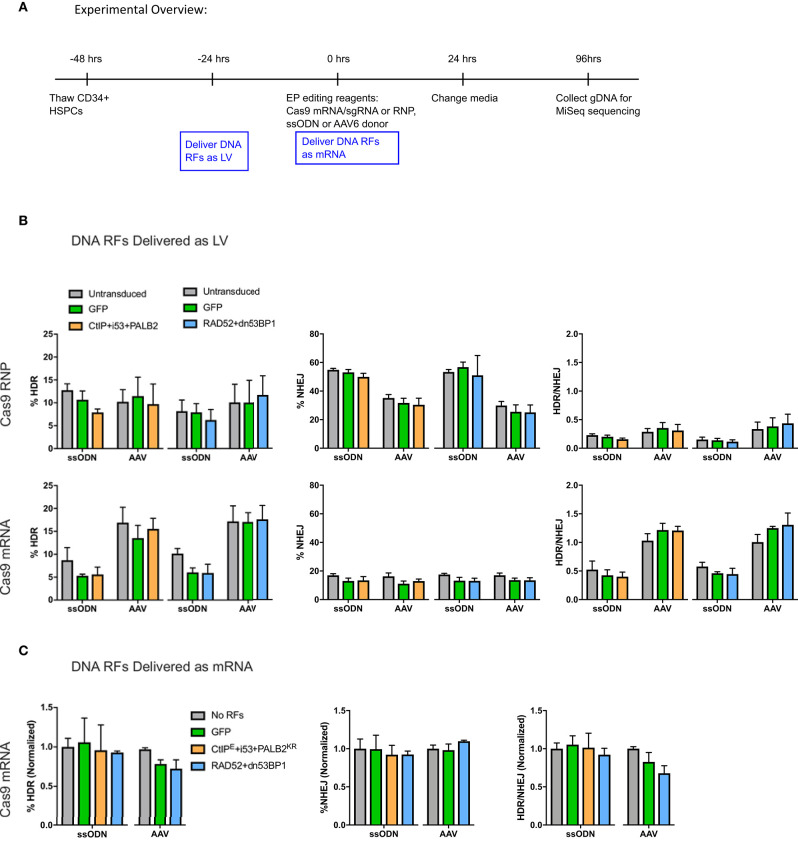
**(A)** Experimental overview of CD34^+^ HSPC transduction and electroporation of DNA RFs and editing reagents. DNA RFs were co-expressed with the gene editing reagents. Cas9 nuclease was delivered either as RNP (100 pmol Cas9 protein + 4.5 μg of IVT sgRNA) or IVT Cas9 mRNA (5 μg) + IVT sgRNA (5 μg); donor template was delivered either as AAV6 (MOI 2e4) or ssODN (3 μg). HDR and NHEJ levels were measured by HTS four days post-electroporation. HDR/NHEJ ratio was calculated. **(B)** DNA repair factors delivered as LV 24 h prior to electroporation of editing reagents. n = 4 biological replicates for all CtIPDE+PALB2KR+i53 LV experiments (Cas9 RNP or Cas9mRNA); n = 8–12 for RAD52+dn53BP1 LV experiments using Cas9 RNP, or n = 4 for RAD52+dn53BP1 LV experiments using Cas9 mRNA. Error bars, mean ± SD. Differences are not significant if not specified, based on Wilcoxon rank sum test. **(C)** DNA RFs were co-delivered as IVT mRNA with editing reagents. n = 2–6 biological replicates for all conditions. Data are normalized to “no RFs” conditions for each set of experiments. Error bars, mean ± SD. Differences are not significant if not specified, based on Wilcoxon rank sum test.

To evaluate the effects of DNA RF overexpression on gene editing levels in primary human CD34^+^ HSPCs, DNA RFs were delivered by either LVs or as *in vitro* transcribed (IVT) mRNAs due to the toxicity associated with plasmid electroporation in HSPCs (Hollis et al., 2006). Cas9 endonuclease was delivered as RNP (Cas9 protein + IVT sgRNA) or as mRNA (IVT Cas9 mRNA + IVT sgRNA), and donor template was delivered either as ssODN or AAV6. DNA RF were delivered as LVs to the HSPCs 24 h prior to electroporation, and IVT DNA RF mRNAs were co-electroporated with the gene editing reagents (Figure 2A).

No benefit in HDR or NHEJ levels were observed with the addition of DNA RFs compared to controls irrespective of delivery method (Figures 2B,C). Interestingly, while the levels of HDR achieved with Cas9 RNP and Cas9 mRNA ranged between 5 and 15%, the levels of NHEJ were higher with Cas9 RNP (35–60%) compared to Cas9 mRNA (12–15%). The HDR/NHEJ ratio was lower for all conditions edited with a ssODN compared to an AAV6 donor (Figures 2B,C). These differences in Cas9 nuclease delivery and donor template utilization, although beyond the scope of this study, suggest interesting distinctions in DNA damage repair pathways.

Because prior experiments were performed on unsorted CD34^+^ HSPCs, the effects on gene editing outcomes from overexpression of the DNA RFs CtIP^DE^, PALB2^KR^, and i53, which we hypothesized would initiate HDR in G1 phase of the cell cycle, may have been overlooked (Orthwein et al., 2015). To evaluate whether these DNA RFs improved gene editing outcomes specifically in G0/G1 phases, HSPCs were transduced with the indicated DNA RFs or GFP LVs 24 h prior to fluorescence-activated cell sorting (FACS) into G0/G1 or S/G2/M populations, and the populations in the different cell cycle stages were immediately electroporated with editing reagents.

**Figure 3:**
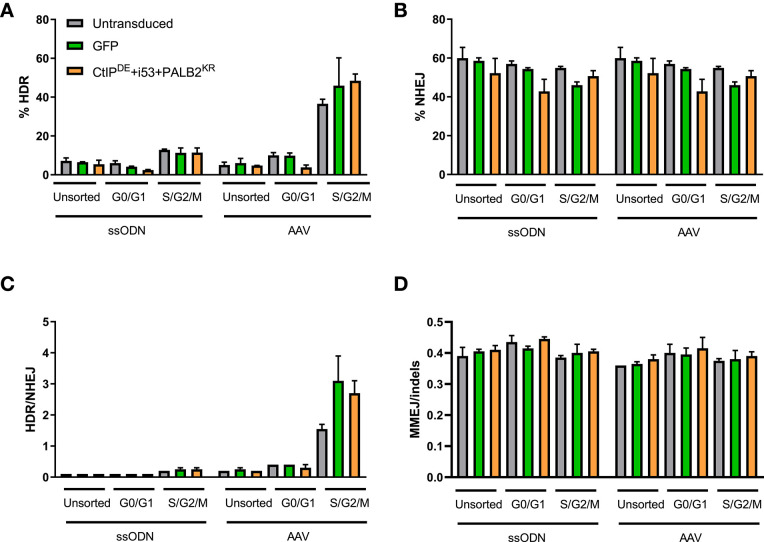
CtIP^DE^+PALB2^KR^ + i53 overexpression in G1 phase of the cell cycle does not activate HDR. **(A–D)** Gene editing levels in sorted populations. Hoescht stain was used to discriminate G0/G1 and S/G2/M phases. Cells were pre-transduced with the indicated DNA RFs or GFP LVs, sorted into cell cycle phases and then electroporated with Cas9 RNP and ssODN or transduced with an AAV6 donor template immediately after electroporation. HDR and NHEJ levels were measured by HTS. **(A)** HDR, **(B)** NHEJ, **(C)** HDR/NHEJ, **(D)** MMEJ/indels, in unsorted, G0/G1-sorted, or S/G2/M-sorted populations. *n* = 2 biological replicates. Error bars, mean ± SD.

As expected, the levels of HDR were higher in the S/G2/M population compared to G0/G1-sorted and unsorted control for all conditions, while the levels of NHEJ were similar across all conditions and cell cycle stages (Figures 3A,B). Of note, while the levels of HDR in unsorted and G0/G1-sorted cells were comparable for both donor templates (5–7%), the levels of HDR in the S/G2/M-sorted cells edited with an AAV6 donor were higher (36–50%) than in cells edited with a ssODN donor (11–13%), resulting in an increased HDR/NHEJ ratio with the AAV6 donor. However, there were no statistically significant differences in the levels of HDR, NHEJ, or MMEJ between cells transduced with the DNA RFs, GFP, or untransduced cells within a cell cycle stage population (Figures 3A–D). There was a slight increase in the HDR/NHEJ ratio in cells transduced with the DNA RFs (1.7-fold) or GFP (2-fold) relative to untransduced cells within the S/G2/M population edited with an AAV6 donor (Figure 3C). These data suggest that overexpression of CtIP^DE^, PALB2^KR^ and i53 did not result in improved gene editing outcomes in G0/G1 cell cycle phase.

### Evaluating a Panel of Cas9 Fusion Variants to Promote HDR or Decrease NHEJ

As previously stated, global, albeit transient, manipulation of DNA RFs may pose a threat to genome integrity (Jayavaradhan et al., 2019). Although extensive toxicity due to global overexpression of DNA RFs was not seen in this work (data not shown), no improvement in gene editing was observed either when these RFs were over-expressed, either stably (LV) or transiently (IVT mRNA). As an alternative approach to deliver these DNA RFs to the site of the Cas9-induced DSB, we have produced a series of novel Cas9 fusion proteins by adding sequences encoding proteins that may modulate DNA repair pathways by promoting HDR or inhibiting NHEJ (Table 2). One set of fusions contained the HDR enhancer element of hCtIP (Cas9-hCtIP; Charpentier et al., 2018). CtIP is necessary for DSB resection to generate single stranded-DNA (ssDNA), required for homology searching and strand invasion, and therefore is required for homologous recombination (HR). We have made modifications to this Cas9 variant by adding a flexible linker between the C-terminus of Cas9 and the N-terminus of the hCtIP fragment (Supplementary Figure 3A). The Cas9-GSG-CtIP variant contains a 12 amino acid linker made up of repeating Gly-Ser-Gly residues (GGGS)×3. Cas9-TGS-CtIP contains a 12 amino acid linker made up of repeating Tyr-Gly-Ser residues (TGS)×4. Moreover, we have constructed a double fusion variant containing a fragment of the hGem protein fused between Cas9 and the hCtIP fragment (Cas9-hGem-hCtIP).

**Table 2:** Panel of Cas9-fusion variants assessed in this study.

**Cas9 variant**		**Description**
Cas9	Cas9	Wild-type Cas9
Cas9-hGem	Cas9-hGem	hGeminin is degraded by the APC/Cdh1 complex during G1 phase when the NHEJ pathway is selectively active over HDR (Gutschner et al., 2016)
Cas9-hCtIP	Cas9-hCtIP Cas9-GSG-CtIP^*^ Cas9-TGS-CtIP^**^	hCtIP “HDR enhancer element” is involved in the DNA end resection. Involved in recruiting other factors to initiate repair (Charpentier et al., 2018).
	Cas9-hGem-hCtIP	
Cas9-UL12	Cas9-UL12	UL12 increases recombination by the single-strand annealing (SSA) pathway and inhibits NHEJ (Balasubramanian et al., 2010).
	Cas9-hGem-UL12	
Cas9-dn53BP1	Cas9-dn53BP1	A mouse dominant negative 53BP1 is expected to reduce accumulation of 53BP1 at the DSB site, thus suppressing NHEJ (Paulsen et al., 2017)
	Cas9-DN1S Cas9-GSG-DN1S^*^	Amino acids 1,231–1,644 of human 53BP1; (Jayavaradhan et al., 2019)

* *GSG—signifies a 12 amino acid linker made up of repeating Gly-Ser-Gly residues (GGGS)×3*.

** *TGS—signifies a 12 amino acid linker made up of repeating Tyr-Gly-Ser residues (TGS)×4*.

We have also constructed a Cas9 variant which contains a 126 amino acid N-terminal fragment from the Herpes Simplex Virus protein UL12, fused to the C-terminus of Cas9 (Reuven et al., 2019). UL12 may recruit subsets of the critical HDR complex proteins to the nuclease-mediated cleavage site, increasing the yield of HDR–mediated editing outcomes. We have made subsequent modifications to the Cas9-UL12 variant by adding the hGem fragment (Cas9-hGem-UL12).

The Cas9-dn53BP1 variant contains a fragment of the mouse 53BP1, a DNA repair protein involved in the recruitment of NHEJ factors to a DSB, fused to Cas9. Previous reports have shown that global transient expression of dn53BP1 in cell lines can decrease NHEJ. We have fused this fragment to the C-terminus of Cas9 to assess its ability to block the recruitment of 53BP1 specifically at a Cas9-induced break site. We have tested other dominant negative 53BP1 Cas9 fusion variants, namely Cas9-DN1S (Jayavaradhan et al., 2019; Supplementary Figure 3B). To date, this Cas9-variant has only been assessed in cell lines.

### Editing in a K562 BFP Reporter Cell Line for Preliminary Assessment of Cas9 Variants

**Figure 4:**
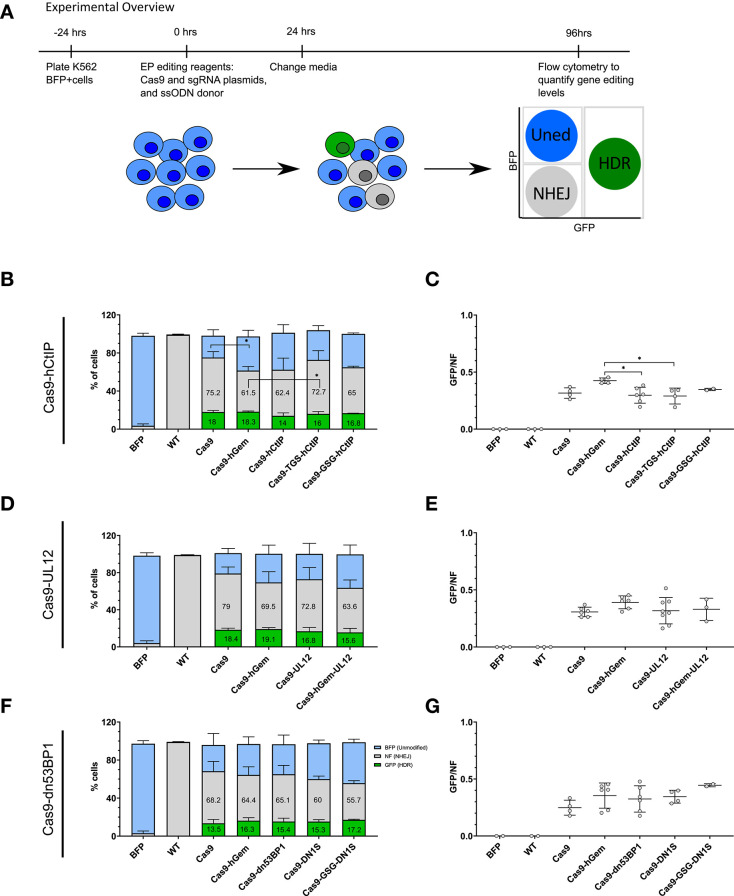
Preliminary assessment of Cas9 variants to modulate local gene editing outcomes in a K562 BFP reporter cell line. **(A)** Experimental overview of electroporation of Cas9 variants as plasmid into a K562 BFP reporter cell line. K562 BFP cells were electroporated with 1 μg Cas9 variant plasmid, 1 μg of sgRNA plasmid targeting the BFP gene, and a ssODN donor (3 μM). Cells were cultured for 4 days post-electroporation prior to analysis by flow cytometry. **(B–G)** Proportion of GFP^+^, BFP^+^, or NF cells and GFP/NF ratio of cells edited with Cas9-hCtIP variants, *n* = 2–6 biological replicates **(B,C)**, and Cas9-UL12 variants, *n* = 3–8 biological replicates **(D,E)**, and Cas9-dn53BP1, *n* = 2–6 biological replicates **(F,G)**. Error bars, mean ± SD. Differences are not significant if not specified, *p < 0.05, based on Wilcoxon rank sum test.

To initially screen a panel of these novel Cas9 fusion variants, as well as the fusion of Cas9 to a fragment from hGem to destabilize Cas9 in the G1 phase as we previously described (Lomova et al., 2019), for their ability to promote HDR or limit NHEJ, the sequences encoding these Cas9 fusion proteins were cloned into MND-LTR-U3-expression plasmids. These were co-electroporated with a plasmid encoding a sgRNA targeting a stably integrated monoallelic BFP reporter gene in a K562 cell line (Richardson et al., 2018; Figure 4A). Cas9 editing at the BFP locus results in either disruption of the BFP gene by NHEJ or modification to the eGFP gene by HDR, depending on the activated DNA repair pathway and presence of a donor template. Formation of either in-frame or frameshift indels by the NHEJ pathway at the target site will result in disruption of the BFP gene, resulting in non-fluorescent cells [BFP^−^/GFP^−^; non-fluorescent {NF}; Glaser et al., 2016]. The addition of a ssODN donor template containing a single point mutation that alters the 66th amino acid of the BFP gene from a histidine to a tyrosine results in conversion of the BFP gene to eGFP upon HDR (BFP^−^/GFP^+^; “GFP”). The donor also contains an additional single nucleotide polymorphism (SNP) at the PAM recognition site to prevent re-cleavage by the Cas9 nuclease of the HDR-edited sequence. Unedited cells will remain BFP^+^/GFP^−^ (“BFP”).

Preliminary comparison of the Cas9 fusion variants by phenotypic assessment of edited cells using flow cytometry resulted in baseline wild-type Cas9 editing of 68.2–79% NF cells (NHEJ) and 13.5–18.4% GFP^+^ (HDR) cells. Editing with the Cas9-hGem fusion resulted in slightly reduced gene disruption (61.5–69.5%), and similar levels of GFP^+^ cells as with wild-type Cas9 (16.3–19.1%; Figures 4B,D,F). We have previously reported that the Cas9-hGem fusion reduces NHEJ by 50% in primary human HSPCs at the *HBB* locus (Lomova et al., 2019). We believe that the limited decrease in NHEJ with Cas9-hGem seen using this K562 BFP reporter assay is due to the differences in cell cycle distribution of K562 cells relative to HSPCs (Supplementary Figure 4).

Among the Cas9-hCtIP variants tested, editing with the Cas9-hCtIP and the Cas9-GSG-hCtIP fusion proteins resulted in a ~15% reduction of NF cells (NHEJ), with a slight reduction in GFP^+^ cells compared to Cas9 editing. Cas9-TGS-hCtIP had a similar editing profile to Cas9 alone (Figure 4B). In this reporter system, the GFP/NF ratio is used to estimate the HDR/NHEJ ratio. Cas9-hCtIP and Cas9-TGS-CtIP has significantly reduced GFP/NF ratios compared to Cas9-hGem (Figure 4C).

Among the Cas9-UL12 variants tested, the Cas9-UL12 fusion did not alter repair pathway choice compared to Cas9 or Cas9-hGem. Interestingly, editing with the Cas9-hGem-UL12 fusion led to a 20% decrease in BFP disruption (63.6% compared to 79%), with a modest decline in HDR levels compared to Cas9 (15.6% compared to 18.4%; Figure 4D). However, the GFP/NF ratio was not significantly different among these Cas9-UL12 variants compared to Cas9 or Cas9-hGem (Figure 4E).

Among the Cas9-dn53BP1 variants tested, all variants reduced the percentage of resulting NF cells compared to Cas9. Cas9-dn53BP1 editing resulted in a 4.5% relative decrease in NF cells (65.1% compared to 68.2%), while maintaining the level of GFP^+^ cells. Cas9-DN1S and Cas9-GSG-DN1S editing resulted in a 12 and 18.3% reduction of NF cells, respectively, compared to Cas9 alone. Cas9-GSG-DN1S had similar levels of GFP^+^ cells to Cas9-hGem, with a 13.5% reduction in NF cells (55.7% compared to 69.2%). These findings suggest that Cas9-DN1S and Cas9-GSG-DN1S are the most effective variants at reducing NHEJ compared to Cas9 and Cas9-hGem when editing cell lines. (Figures 4F,G).

### Assessing Cas9 Variant Editing in Primary Human CD34^+^ HSPCs

**Figure 5:**
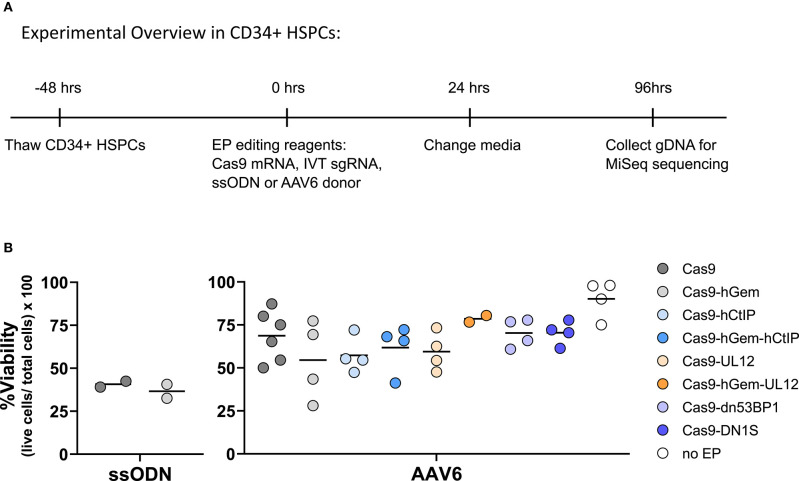
Experimental outline of Cas9 variant gene editing in HSPCs. **(A)** Experimental overview of electroporation of Cas9 variants delivered as IVT mRNA to CD34^+^ HSPCs. Cas9 variants were electroporated at equimolar amounts (3 pmol) with 120 pmol of IVT sgRNA targeting the SCD mutation at the *HBB* locus, and either a ssODN (3 μM) or AAV6 donor (MOI 2e4). Editing outcomes were measured by MiSeq HTS 4 days post-electroporation. **(B)** Viability of CD34^+^ HSPCs edited with Cas9 variants, and an ssODN or AAV6 donor 24 h post-electroporation. *n* = 2–6 biological replicates. Center line represents mean. Differences are not significant if not specified, based on Wilcoxon rank sum test.

Following preliminary assessment of the Cas9 variants in a K562 BFP reporter cell line, a subset of Cas9 variants [Cas9-hGem, Cas9-hCtIP, Cas9-hGem-hCtIP, Cas9-UL12, Cas9-hGem-UL12, Cas9-dn53BP1, Cas9-DN1S] were tested in primary human CD34^+^ HSPCs by targeted editing of the SCD causative mutation at the *HBB* locus. HSPCs were edited with IVT Cas9 mRNA and IVT sgRNA targeting exon 1 of the *HBB* locus, along with a ssODN or AAV6 donor conferring modifications at the site of the sickle mutation and the PAM site (Figure 5A). Since the Cas9 variant transcripts vary in length (4–6 kb), going forward, we modified the protocol to test these variants at equimolar amounts rather than equal weight (Supplementary Figures 5A,B). Editing outcomes were assessed by HTS of the *HBB* target site. Viability of HSPCs at 24 h post-electroporation was unaffected by the Cas9 variants compared to Cas9 or Cas9-hGem (Figure 5B).

**Figure 6:**
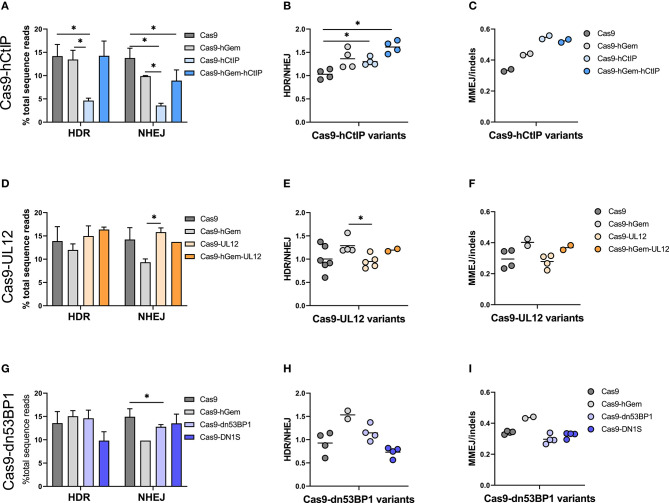
Cas9 variant gene editing of the β-globin locus with an AAV6 donor reveals distinctive DNA repair outcomes in HSPCs. CD34^+^ HSPCs edited with Cas9 variants (3 pmol Cas9 mRNA + 120 pmol IVT sgRNA) and an AAV6 donor (MOI 2e4). Editing outcomes were measured by MiSeq HTS 4 days post-electroporation. Cas9 and Cas9-hGem were used as controls. **(A–C)** Gene editing outcomes with Cas9-hCtIP variants. **(A)** HDR and NHEJ. *n* = 4 biological replicates). Error bars, mean ± SD. **(B)** HDR/NHEJ. *n* = 4 biological replicates Center line represents mean **(C)** MMEJ/indels. *n* = 2 biological replicates. Center line represents mean. **(D–F)** Gene editing outcomes with Cas9-UL12 variants. **(D)** HDR and NHEJ. *n* = 2–6 biological replicates. Error bars, mean ± SD. **(E)** HDR/NHEJ. *n* = 2–6 biological replicates Center line represents mean **(F)** MMEJ/indels. *n* = 2–4 biological replicates. Center line represents mean. **(G–I)** Gene editing outcomes with Cas9-dn53BP1 variants. **(G)** HDR and NHEJ. *n* = 2–4 biological replicates. Error bars, mean ± SD. **(H)** HDR/NHEJ, *n* = 2–4 biological replicates. Center line represents mean **(I)** MMEJ/indels. *n* = 2–4 biological replicates. Center line represents mean. Differences are not significant if not specified, *p < 0.05, based on Wilcoxon rank sum test.

Gene editing with Cas9 mRNA and an AAV6 donor led to ~14% HDR and 15% NHEJ in HSPCs edited with Cas9 on average across all experiments (Figures 6A,D,G). Cas9-hGem editing with the AAV6 donor maintained levels of HDR and decreased the frequency of NHEJ by one third compared to Cas9 (10 vs. 15%, respectively), as previously reported (Lomova et al., 2019).

Editing by the Cas9-hCtIP was consistently low, presumably due to reduced nuclease activity compared to Cas9; this was partially rescued by the addition of the hGem fragment between Cas9 and hCtIP (Cas9-hGem-hCtIP). Interestingly, while the hGem-hCtIP double fusion did not result in an increase in HDR relative to Cas9 or Cas9-hGem, similar levels of NHEJ were achieved between the Cas9-hGem-hCtIP and Cas9-hGem variants (Figure 6A). There was consistently significantly improved HDR/NHEJ ratio for variants containing hGem compared to Cas9 alone (Cas9-hGem, Cas9-hGem-hCtIP; Figure 6B).

CtIP has been implicated in stimulating significant MMEJ, an HDR-mediated event that leads to specific sized indels, in the presence of homologous sequences flanking a DSB. We have noted a frequent 9 base pair deletion in *HBB* among the indels around the Cas9-induced DSB that is presumed to be an MMEJ event. When assessing MMEJ out of total indel-forming events (MMEJ/indels), we noted that Cas9 variants containing the hCtIP fragment had higher levels of MMEJ, suggesting that the hCtIP fragment is biologically active but is not inducing HR or SSTR (Figure 6C, Supplementary Figure 6). This finding may be valuable for targeted gene editing in which the end goal is to induce a specific MMEJ-mediated deletion (Métais et al., 2019).

When comparing Cas9 variants containing a UL12 fusion in the context of an AAV6 donor, there were no remarkable differences in HDR or NHEJ by either Cas9-UL12 or Cas9-hGem-UL12 relative to Cas9, while Cas9-UL12 lead to significantly higher NHEJ than Cas9-hGem (Figure 6D). Cas9-UL12 had a similar HDR/NHEJ ratio to Cas9 alone, while Cas9-hGem-UL12 had a similar HDR/NHEJ ratio to Cas9-hGem, suggesting that the hGem fragment, and not UL12, is driving these differences (Figure 6E). The ratios of MMEJ/indels were not different between Cas9 and Cas9-UL12, or Cas9-hGem and Cas9-hGem-UL12 (Figure 6F).

Among the dn53BP1 variants tested, Cas9-dn53BP1 resulted in significantly reduced NHEJ levels, compared to Cas9, but still higher than Cas9-hGem (Figure 6G). Editing with Cas9-DN1S using an AAV6 donor resulted in decreased levels of HDR (10% from 14%) while similar levels of NHEJ relative to Cas9, contrary to what was seen previously in this work and in previous reports in cell lines (Figure 6G; Jayavaradhan et al., 2019). Cas9-DN1S editing resulted in a decreased HDR/NHEJ ratio compared to Cas9 and Cas9-hGem (Figure 6H). No differences in MMEJ/indels ratios were observed with the Cas9-dn53BP1 variants (Figure 6I).

**Figure 7:**
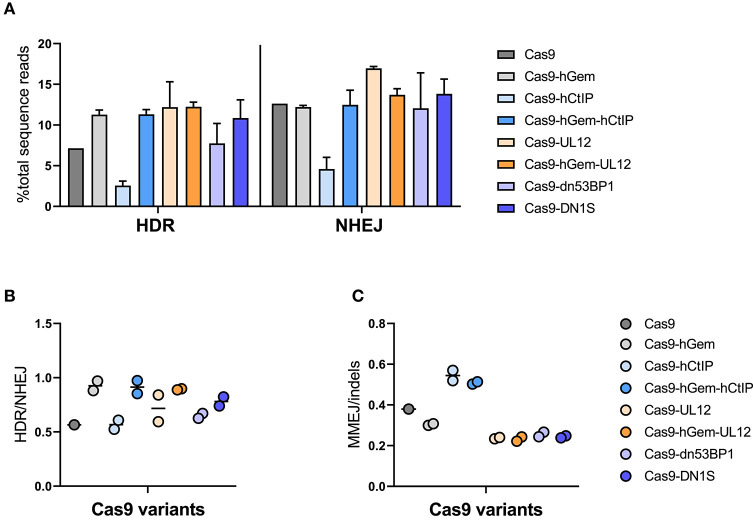
Cas9 variant gene editing of the *HBB* locus with a ssODN donor. CD34^+^ HSPCs edited with Cas9 variants (3 pmol Cas9 mRNA + 120 pmol IVT sgRNA) and ssODN donor (3 μM). Editing outcomes were measured by MiSeq HTS 4 days post-electroporation. Cas9 and Cas9-hGem were used as controls. **(A)** HDR and NHEJ. **(B)** HDR/NHEJ *n* = 2 biological replicates. Error bars, mean ± SD. **(C)** MMEJ/indels. *n* = 2 biological replicates. Center line represents mean.

Gene editing with Cas9 mRNA and a ssODN donor resulted in ~7% HDR and 13% NHEJ in human HSPCs, while Cas9-hGem editing lead to a slight increase in HDR (10%) with no reduction in NHEJ (11%; Figure 7A). As previously reported, Cas9-hCtIP editing appeared impaired and resulted in lower levels of HDR and NHEJ relative to Cas9 and Cas9-hGem. However, the addition of the hGem fragment to Cas9-hCtIP improved nuclease activity similar to Cas9-hGem levels. Levels of HDR did not change for the Cas9-UL12 variants when compared to Cas9-hGem in these experiments; however, Cas9-UL12 editing did result in an increase in NHEJ (from ~12.5 to ~17%), suggesting that the addition of UL12 may be promoting exonuclease activity (as described in Schumacher et al., 2012), but expression of UL12 alone may not be sufficient to promote HDR with a ssODN donor. Among the Cas9-dn53BP1 variants tested, there was a slight decrease in HDR with Cas9-dn53BP1 compared to Cas9-hGem, falling to similar levels as Cas9 alone. Interestingly, editing with Cas9-DN1S did not reduce NHEJ in the context of editing primary HSPCs with a ssODN donor (Figure 7A).

**Figure 8:**
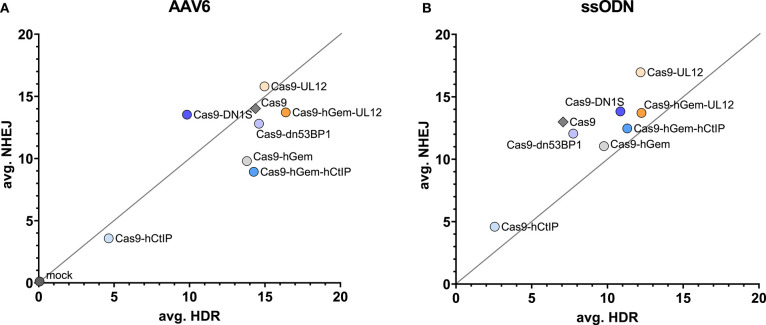
Comparison of averageCas9 variant editing in HSPCs with a ssODN or AAV6 donor. **(A)** Average HDR vs. average NHEJ for all Cas9 variants assessed in this study. The *y* axis reflects average NHEJ editing, and the *x* axis reflects the average HDR editing of each variant at the *HBB* locus. Average editing of Cas9 variants targeting the SCD mutation in CD34^+^ HSPCs with an AAV6 donor **(A)** or an ssODN donor **(B)**.

Overall, the HDR/NHEJ ratio increased relative to Cas9 for all variants containing the hGem fragment; there was no further improvement to the HDR/NHEJ ratio by the additional fusion proteins (Figure 7B). All variants containing the CtIP fragment had increased MMEJ/indel ratios relative to Cas9 and Cas9-hGem (Figure 7C). In summary, the Cas9 variants containing hGem had the most favorable HDR/NHEJ ratios irrespective of donor template type (AAV6 or ssODN; Figures 8A,B).

## Discussion

In this study, we investigated whether gene editing outcomes can be modified by manipulating DNA repair pathways. Specifically, we aimed to increase the frequency of HDR and/or reduce NHEJ-mediated repair by global and local manipulation of endogenous DNA repair pathways in human hematopoietic stem and progenitor cells. A panel of DNA RFs that have been shown to promote HDR or inhibit NHEJ in various cell lines were assessed for their ability to manipulate gene editing outcomes in K562 cells and in primary human CD34^+^ HSPCs, when co-expressed with editing. To synchronize DNA RF expression to the time of Cas9-induced DSB and DNA repair, K562s were pre-transduced with LVs expressing DNA RFs. Constitutive overexpression of the DNA RFs was demonstrated by western blots. However, the expressed DNA RFs had no effect on HDR or NHEJ levels when cells were edited with Cas9 plasmid or Cas9 RNP targeting the site of the SCD mutation at the *HBB* locus relative to cells that were untransduced or transduced with a GFP control LV.

Similar gene editing trends were seen when DNA RFs were globally overexpressed in CD34^+^ HSPCs edited with Cas9 mRNA or Cas9 RNP targeting the *HBB* locus. Delivery of the DNA RFs as either constitutively expressed LVs or transiently expressed mRNA did not alter HDR or NHEJ relative to “no RFs” or GFP control conditions. This signified that the combination of DNA RFs used in this study was unable to manipulate endogenous DNA repair to favor HDR over NHEJ.

We hypothesized that the combination of CtIP^DE^ + PALB2^KR^ + i53 DNA, would specifically promote HDR in the G1 phase of the cell cycle. Sequencing of editing outcomes in bulk (unsynchronized) HSPCs may mask this phenomenon. To overcome this, we sorted pre-transduced HSPCs that expressed the various factors of interest immediately prior to gene editing into different cell cycle populations, and assessed editing outcomes in the G0/G1 and S/G2/M sorted populations. As expected, levels of HDR were higher in the S/G2/M sorted population relative to G0/G1 or unsorted populations, as HDR is selectively active in S/G2 phases of the cell cycle. However, no further improvement in HDR was seen with the expression of combined CtIP^DE^ + PALB2^KR^ + i53, suggesting that the expression of these combination of factors alone is not enough to manipulate DNA repair pathways in primary HSPCs.

In a parallel approach, we assessed how localization of DNA RFs to the Cas9-induced DSB site by fusing DNA RFs directly to the C- terminus of Cas9 would affect gene editing outcomes. We tested a panel of Cas9 fusion protein variants for their ability to promote HDR or to inhibit NHEJ initially in a K562 BFP reporter cell line, then in primary human HSPCs. As previously reported, editing HSPCs with Cas9-hGem and an AAV6 consistently improved the HDR/NHEJ ratio compared to Cas9 editing, predominantly by a decrease in NHEJ alleles (Lomova et al., 2019). A similar increase in the HDR/NHEJ ratio was seen with Cas9-hGem editing and a ssODN donor; however, these results seem to be driven by an increase in HDR, rather than a decrease in NHEJ. An increase in the MMEJ/indels ratio was also seen in Cas9-hGem edited cells.

Cas9-hCtIP editing in K562 BFP cells was comparable to Cas9 editing; however, Cas9-hCtIP nuclease activity was severely impaired in the context of HSPCs gene editing. This may suggest differential DNA repair states between K562 cells and primary HSPCs, where CtIP expression during an HDR non-permissive state may impair canonical HDR and NHEJ repair systems. Gene editing by Cas9-hGem-hCtIP had a similar profile to Cas9-hGem when assessing HDR, NHEJ, and the HDR/NHEJ ratio. However, both CtIP-containing Cas9 fusions promoted an increase in MMEJ-mediated repair outcomes, confirming that the CtIP element is biologically active and is able to shift repair toward an error-prone HDR pathway. Cas9-UL12 editing resulted in higher NHEJ relative to Cas9 and Cas9-hGem when a ssODN donor was used. The N-terminal domain of UL12 used in this study is sufficient to recruit the MRN complex, a vital step toward HDR (Reuven et al., 2019). However, it does not possess exonuclease activity which may further stimulate the production of 3′ overhangs and have a stronger influence on shifting repair outcomes toward HDR. Cas9-DN1S, expressing dominant negative 53BP1 fragment, effectively decreased the frequency of NHEJ in K562 cells, but did not have a similar effect in primary HSPCs.

Interestingly, we noted that HDR levels increased in K562 cells that were edited using a Cas9/sgRNA plasmid and DNA RFs or GFP expressed as plasmid, suggesting that the increase in editing is not directly related to the DNA RFs but to plasmid co-delivery. No increase in HDR was seen when K562 cells were edited with Cas9 RNP and plasmid DNA RFs. A recent report suggests that co-transfection of large plasmid cassettes with small plasmid vectors (3 kb) can improve transfection efficiency and viability of cell lines and primary cell types (Søndergaard et al., 2020). These findings could explain our observation of increased editing in K562 cells only when Cas9 plasmid was used in combination with DNA RF or GFP plasmids. However, other potential hypotheses remain and need to be tested; plasmid electroporation may increase transcription and/or translation of Cas9 plasmid, thus increasing total Cas9 activity and thereby increasing HDR, or plasmid electroporation may enhance the DNA damage response in K562 cells, enhancing DNA repair pathways and increasing HDR and NHEJ levels in the cells.

Overall, this work underlines the complexity of DNA repair regulation and the challenges to harnessing it to achieve curative gene editing levels in therapeutically relevant cell types. The consistent performance of the Cas9 fusions with the fragment of human Geminin domain to improve the ratio of HDR to NHEJ events strongly supports further evaluation of this variant for potential clinical applications. The ability of the CtIP fusions to promote MMEJ may also have specific indications. Continued efforts to successfully manipulate DNA repair pathways may lead to improved methods of gene editing for gene therapy.

## Materials and Methods

### K562 Cells

K562 cells were modified to contain sickle cell disease-causing mutation, as described previously (Hoban et al., 2016). K562 BFP cells were modified to contain monoallelic copy of the BFP gene, as described in Richardson et al. (2016).

### Primary Human CD34^+^ Cells

Leukopaks from healthy donors were purchased from HemaCare (HemaCare BioResearch Products; Van Nuys, CA). Mobilized peripheral blood (mPB) was collected from normal, healthy donors on days 5 and 6 after 5 days of stimulation with granulocyte-colony stimulating factor (G-CSF). Briefly, leukapheresis bags were washed three times with PBS/EDTA at room temperature (RT) and spun down at 150×g. Platelet depletion was performed from the centrifuged bags at each wash step using a plasma expressor extractor (Fenwal). The subsequent enrichment of CD34^+^ cells was done by using the CliniMACS Plus (Miltenyi; Bergish Gladbach, Germany). Cells were cryopreserved in CryoStor CS5 (Stemcell Technologies; Vancouver, Canada) using a CryoMed controlled-rate freezer (Thermo Fisher Scientific; Waltham, MA).

### Cell Culture

K562 cells were cultured in RPMI medium + 10% heat-inactivated fetal bovine serum [HI FBS (Gibco/ThermoFisher; Waltham, MA)] + 1% penicillin, streptomycin, glutamine [PSQ (Gemini Bio-Products; Sacramento, CA)], and were kept at a density between 1 × 10^5^ and 1 × 10^6^ cells per ml. Healthy human CD34^+^ cells from mPB (peripheral blood stem cells, PBSCs) were thawed in pre-warmed X-Vivo 15 medium (Lonza; Basel, Switzerland) with 1% PSQ, pelleted at 500×g for 5 min, and resuspended at 5 × 10^5^ cells/mL in pre-warmed X-Vivo 15 medium with PSQ and SFT cytokines [50 ng/mL stem cell factor (SCF), 50 ng/mL fms-related tyrosine kinase 3 ligand (Flt3-L), and 50 ng/mL thrombopoietin (TPO)] (Peprotech; Rocky Hill, NJ). Cells were pre-stimulated at 37°C and 5% CO_2_ incubator for 48 h.

### LV/IDLV Transduction

To deliver LV/IDLV DNA RFs, cells were transduced with the MOIs indicated in figure legends for 24 h (additional time points were tested, but data not shown). Transduction enhancers (PGE2 and Poloxamer Synperonic F108) were added during transduction, as described elsewhere (Masiuk et al., 2019).

### K562 Cell Electroporation With DNA RFs

K562 cells were split 1:5 1 day before the electroporation. Where indicated, the cells were transduced with LV or IDLV 24 h prior to electroporation. On the day of electroporation, the cells were counted on ViCell (Beckman Coulter; Brea, CA), 2 × 10^5^ cells per condition were centrifuged at 90×g for 15 min at RT, resuspended in 20 μl of SF electroporation buffer (Lonza; Basel, Switzerland), combined with Cas9 plasmid or RNP, 3 μM ssODN (where applicable), and DNA RF or GFP plasmids (where applicable). The cells were electroporated on Amaxa 4D Nucleofector X Unit (Lonza; Basel, Switzerland) using FF-120 setting. After electroporation, the cells were rested in electroporation strips for 10 min at RT, and then recovered with 500 μl of RPMI medium + 10% HI FBS (Gibco/ThermoFisher; Waltham, MA) + 1% PSQ (Gemini BioProducts; Sacramento, CA). AAV6 donor template was added to recovery medium where applicable. Twenty-four hours post electroporation, the cells were re-plated into fresh medium. The cells were harvested 4 days post electroporation for gDNA extraction to evaluate gene editing levels. gDNA was extracted using PureLink Genomic DNA Mini Kit (Invitrogen/ThermoFisher Scientific; Carlsbad, CA).

### CD34^+^ HSPC Cell Electroporation With DNA RFs

For electroporation, 2 × 10^5^ (or 1 × 10^6^ for FACS experiment) cells per condition were pelleted at 90×g for 15 min at RT, resuspended in 100 μl of BTXpress Electroporation buffer (Harvard Bioscience, Inc; Holliston, MA), combined with pre-aliquoted ssODN (where applicable), RNP (100 pmol Cas9 protein + 4.5 μg of IVT sgRNA) or 5 μg Cas9 mRNA + 5 μg of IVT sgRNA, kept on ice, and pulsed once at 250 V for 5 ms in the BTX ECM 830 Square Wave Electroporator (Harvard Apparatus; Holliston, MA). After electroporation, cells were rested in cuvettes for 10 min at RT, and then recovered with 400 μl (or 2.4 mL, for 1 × 10^6^ cells) of X-Vivo 15 medium (with PSQ and SFT cytokines). Where applicable, recovery media contained AAV6 (multiplicity of infection, MOI = 2e4) to introduce 4 SNPs (Virovek; Hayward, CA). The cells were cultured in a 24-well (or 6-well, for 1 × 10^6^ cells) plate at 37°C, 5% CO_2_ incubator. Twenty-four hours post electroporation, the cells were diluted 1:2 with trypan blue and counted manually using a hemocytometer to determine viability (number of live cells/number of total cells × 100) and fold expansion (number of cells 24 h after electroporation/number of cells before electroporation). Cells were re-plated into 1 mL (or 5 mL, for 1 × 10^6^ cells) of myeloid expansion medium [Iscove's Modified Dulbecco's Medium (IMDM, Thermo Fisher Scientific; Waltham, MA) + 20% FBS (HI FBS, Gibco/ThermoFisher; Waltham, MA) + 5 ng/mL Interleukin 3 (IL3), 10 ng/mL Interleukin 6 (IL6), 25 ng/mL SCF (Peprotech; Rocky Hill, NJ)], and cultured for 4 days prior to harvesting for genomic DNA (gDNA). gDNA was extracted using PureLink Genomic DNA Mini Kit (Invitrogen/ThermoFisher Scientific; Carlsbad, CA).

### Determination of Vector Copy Number (VCN)

VCN was evaluating using Psi and SDC4 primers as described previously (Masiuk et al., 2019).

### mRNA/sgRNA Production

To make mRNA template, maxi-prepped expression plasmids were linearized with SpeI (NEB; Ipswitch, MA), and purified using PCR purification kit according to manufacturer's protocol. *In vitro* transcription was carried out using mMessage Machine T7 Ultra Transcription Kit (ThermoFisher Scientific; Waltham, MA). mRNA product was purified using the RNeasy MinElute Cleanup Kit (Qiagen; Valencia, CA) following the manufacturer's protocol.

sgRNA template was prepared as previously described (dx.doi.org/10.17504/protocols.io.hdrb256). RNA was purified using the RNeasy MinElute Cleanup Kit (Qiagen; Valencia, CA) following manufacturer's protocol.

### DNA RF and Cas9 Variant Production

DNA RF sequences were cloned into pCCL-MNDU3 (Logan et al., 2004) or pT7 plasmids using Gibson Assembly Cloning Kit (NEB; Ipswich, MA). Gene blocks were ordered from IDT to include homology arms for NEBuilder cloning.

### Flow Cytometry/Fluorescence-Activated Cell Sorting (FACS)

All flow cytometry analysis and FACS were performed on the following instruments: BD LSRII, BD LSRFortessa, BD FACS Aria II, all with the similar 5-laser configurations: UV 355 nm, Violet 405 nm, Blue 488 nm, Yel-Grn 561 nm, Red 633 nm.

### Cell Cycle

Cell cycle FACS was performed as described previously (Lomova et al., 2019). Briefly, CD34^+^ cells were cultured at 5 × 10^5^-1 × 10^6^ cells/mL and stained with 5 μg/mL Hoechst 33342 for 45–60 min at 37°C. Cells were washed with PBS + 2% HI FBS and resuspended at 5 × 10^6^ cells/mL in X-Vivo 15 + 5 μg/mL Hoechst 33342. Cells were sorted into G0/G1 or S/G2/M populations and recovered in X-Vivo15 medium. Immediately after sort, cells were counted, centrifuged at 90×g and electroporated.

### K562 BFP Cell Electroporation and Gene Editing Assessment With Cas9 variants

K562 BFP cells were split 1:5 1 day before the electroporation. On the day of electroporation, the cells were counted on ViCell (Beckman Coulter; Brea, CA), 2 × 10^5^ cells per condition were centrifuged at 90×g for 15 min at RT, resuspended in 20 μl of SF electroporation buffer (Lonza; Basel, Switzerland), combined with 1 μg Cas9 plasmid and 3 μM ssODN ultramer donor (GCCACCTACGGCAAGCTGACCCTGAAGTTCATCTGCACCACC GGCAAGCTGCCCGTGCCCTGGCCCACCCTCGTGACCACCCTGACGTACGGCGTGCAGTGCTTCAGCCGCTACCCCGACCACATGA; Integrated DNA Technologies). The cells were electroporated on Amaxa 4D Nucleofector X Unit (Lonza; Basel, Switzerland) using FF-120 setting. After electroporation, the cells were rested in electroporation strips for 10 min at RT, and then recovered with 500 μl of RPMI medium + 10% HI FBS (Gibco/ThermoFisher; Waltham, MA) + 1% PSQ (Gemini BioProducts; Sacramento, CA).Editing outcomes were measured 4 days post-electroporation by flow cytometry. Cells were sorted into BFP^+^GFP^−^ (unedited), BFP^−^GFP^−^ (non-fluorescent, NHEJ) and BFP^−^GFP^+^ (HDR) populations for gene editing outcomes analysis.

### CD34^+^ HSPC Electroporation With Cas9 Variants

For electroporation, 2 × 10^5^ cells per condition were pelleted at 90×g for 15 min at RT, resuspended in 100 μl of BTXpress Electroporation buffer (Harvard Bioscience, Inc; Holliston, MA), combined with pre-aliquoted ssODN (where applicable), Cas9 mRNA (3 pmol) and IVT sgRNA (120 pmol), and pulsed once at 250 V for 5 ms in the BTX ECM 830 Square Wave Electroporator (Harvard Apparatus; Holliston, MA). After electroporation, cells were rested in cuvettes for 10 min at RT, and then recovered with 400 μl of X-Vivo 15 medium (with PSQ and SFT cytokines). If applicable, cells were recovered with media containing AAV6 (multiplicity of infection, MOI = 2e4) to introduce 4 SNPs (Virovek; Hayward, CA). The cells were cultured in a 24-well plate at 37°C, 5% CO_2_ incubator. Twenty-four hours post electroporation, the cells were diluted 1:2 with trypan blue and counted manually using a hemocytometer to determine viability (number of live cells/number of total cells × 100) and fold expansion (number of cells 24 h after electroporation/number of cells before electroporation). Cells were re-plated into 1 mL (or 5 mL, for 1 × 10^6^ cells) of myeloid expansion medium (Iscove's Modified Dulbecco's Medium (IMDM, Thermo Fisher Scientific; Waltham, MA) + 20% FBS [HI FBS, Gibco/ThermoFisher; Waltham, MA) + 5 ng/mL Interleukin 3 (IL3), 10 ng/mL Interleukin 6 (IL6), 25 ng/mL SCF (Peprotech; Rocky Hill, NJ)], and cultured for 4 days prior to harvesting for genomic DNA (gDNA). gDNA was extracted using PureLink Genomic DNA Mini Kit (Invitrogen/ThermoFisher Scientific; Carlsbad, CA).

### Illumina MiSeq Library Preparation

DNA library for HTS was prepared as described previously (Hoban et al., 2015; Lomova et al., 2019). Briefly, an outer PCR was performed on genomic DNA to amplify a 1.1 kb region of interest (using Outer PCR Forward (Fwd) and Reverse (Rev) primers). A second PCR was performed to add a unique index to the PCR product of each sample to be sequenced (read1/read2 and P5/P7 primers). The PCR products with the indexes were mixed at equal concentrations, which was determined by densitometry of the PCR products and analyzed by gel electrophoresis, to create a pooled library. The pooled library was purified twice using AMPure XP beads (Beckman Coulter Inc.; Brea, CA) and then quantified using ddPCR (QX 200; Bio-Rad Laboratories Inc.; Hercules, CA). HTS was performed at UCLA Technology Center for Genomics & Bioinformatics (TCGB) using MiSeq 2 × 150 paired-end reads (Illumina Inc; San Diego, CA). The sequences for all HSPC editing experiments were deposited to NCBI Sequence Read Archive (SRA): **PRJNA672655**.

### Sequencing Analysis and Calculations

Analysis of sequencing data was performed as described elsewhere (Hoban et al., 2015, 2016; Lomova et al., 2019). Percentage of HDR was calculated as the (number of sequence reads containing a sickle change)/(total reads for that sample)^*^100. Percentage of NHEJ was calculated as the frequency of sequence reads containing an insertion or deletion −50/+36 bases around the nuclease cut site. CRISPResso2 was used for visualization of select experimental samples (Clement et al., 2019).

### Statistical Analysis

Summary statistics including mean and standard deviation were calculated and presented in figures for quantitative measures. For experiments with small n, interpretations of the result were mostly descriptive. Statistical tests between experimental group and control group were carried out via Wilcoxon rank sum test to properly account for non-normality of the data. An alpha of 0.05 was chosen as the significance cut-off for two-tailed statistical testing. All statistical analyses were performed using statistical software R Version 4.0.0 (http://www.R-project.org/).

## Author Contributions

EB, AL, ZR, RH, and DK conceived these studies. EB and AL performed the laboratory studies with assistance from LC, DC, PA, SS, KO, JS, RC, NR, and YS. Biostatistical analyses by XW. EB and AL primarily wrote the paper, with assistance from ZR and DK. All authors contributed to the article and approved the submitted version.

## Conflict of Interest

The authors declare that the research was conducted in the absence of any commercial or financial relationships that could be construed as a potential conflict of interest.
