# Supplementary material for: Global and Local Manipulation of DNA Repair Mechanisms to Alter Site-Specific Gene Editing Outcomes in Hematopoietic Stem Cells
Source: Front Genome Ed. 2020 Dec 10;2:601541. doi: 10.3389/fgeed.2020.601541 (PMC8525354; doi:10.3389/fgeed.2020.601541)
Supplement: Supplementary file 1 [file Presentation_1.zip › supp figures correct order/Supplementary Figure 1.PPTX]

## Slide 1
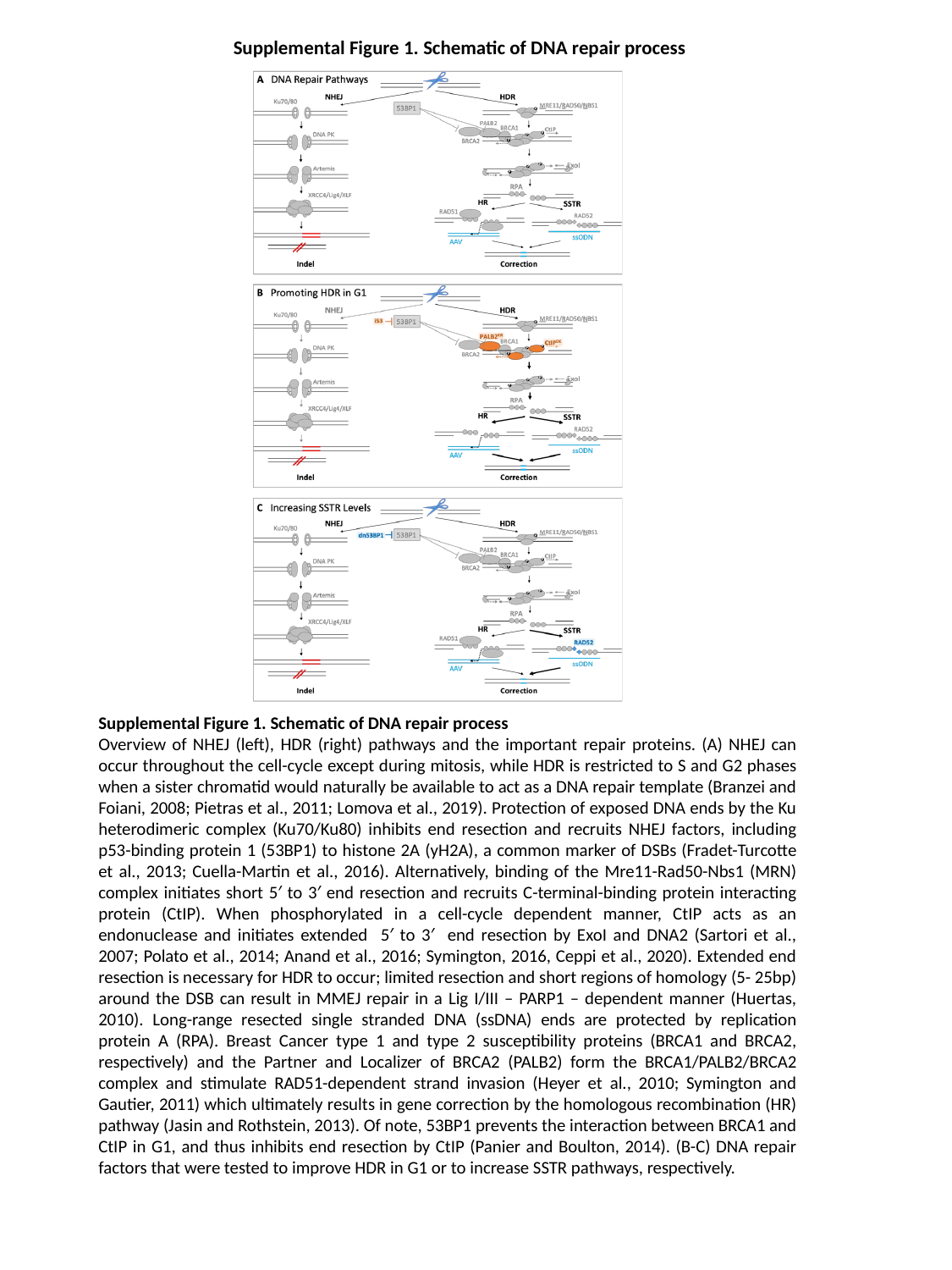

Supplemental Figure 1. Schematic of DNA repair process
Supplemental Figure 1. Schematic of DNA repair process
Overview of NHEJ (left), HDR (right) pathways and the important repair proteins. (A) NHEJ can occur throughout the cell-cycle except during mitosis, while HDR is restricted to S and G2 phases when a sister chromatid would naturally be available to act as a DNA repair template (Branzei and Foiani, 2008; Pietras et al., 2011; Lomova et al., 2019). Protection of exposed DNA ends by the Ku heterodimeric complex (Ku70/Ku80) inhibits end resection and recruits NHEJ factors, including p53-binding protein 1 (53BP1) to histone 2A (yH2A), a common marker of DSBs (Fradet-Turcotte et al., 2013; Cuella-Martin et al., 2016). Alternatively, binding of the Mre11-Rad50-Nbs1 (MRN) complex initiates short 5′ to 3′ end resection and recruits C-terminal-binding protein interacting protein (CtIP). When phosphorylated in a cell-cycle dependent manner, CtIP acts as an endonuclease and initiates extended 5′ to 3′ end resection by ExoI and DNA2 (Sartori et al., 2007; Polato et al., 2014; Anand et al., 2016; Symington, 2016, Ceppi et al., 2020). Extended end resection is necessary for HDR to occur; limited resection and short regions of homology (5- 25bp) around the DSB can result in MMEJ repair in a Lig I/III – PARP1 – dependent manner (Huertas, 2010). Long-range resected single stranded DNA (ssDNA) ends are protected by replication protein A (RPA). Breast Cancer type 1 and type 2 susceptibility proteins (BRCA1 and BRCA2, respectively) and the Partner and Localizer of BRCA2 (PALB2) form the BRCA1/PALB2/BRCA2 complex and stimulate RAD51-dependent strand invasion (Heyer et al., 2010; Symington and Gautier, 2011) which ultimately results in gene correction by the homologous recombination (HR) pathway (Jasin and Rothstein, 2013). Of note, 53BP1 prevents the interaction between BRCA1 and CtIP in G1, and thus inhibits end resection by CtIP (Panier and Boulton, 2014). (B-C) DNA repair factors that were tested to improve HDR in G1 or to increase SSTR pathways, respectively.
