# Supplementary material for: Global and Local Manipulation of DNA Repair Mechanisms to Alter Site-Specific Gene Editing Outcomes in Hematopoietic Stem Cells
Source: Front Genome Ed. 2020 Dec 10;2:601541. doi: 10.3389/fgeed.2020.601541 (PMC8525354; doi:10.3389/fgeed.2020.601541)
Supplement: Supplementary file 1 [file Presentation_1.zip › supp figures correct order/Supplementary Figure 2.PPTX]

## Slide 1
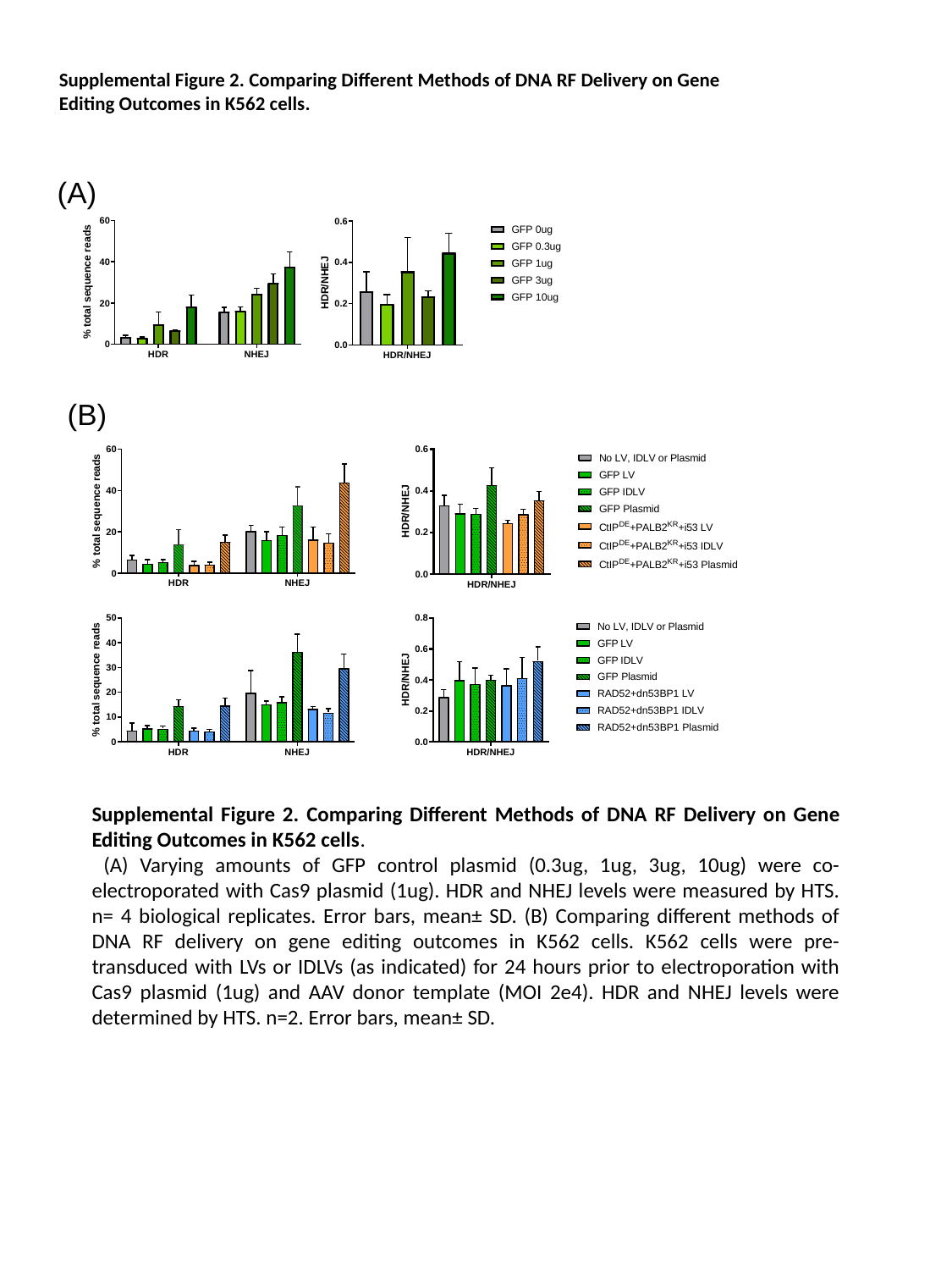

Supplemental Figure 2. Comparing Different Methods of DNA RF Delivery on Gene Editing Outcomes in K562 cells.
Supplemental Figure 2. Comparing Different Methods of DNA RF Delivery on Gene Editing Outcomes in K562 cells.
 (A) Varying amounts of GFP control plasmid (0.3ug, 1ug, 3ug, 10ug) were co-electroporated with Cas9 plasmid (1ug). HDR and NHEJ levels were measured by HTS. n= 4 biological replicates. Error bars, mean± SD. (B) Comparing different methods of DNA RF delivery on gene editing outcomes in K562 cells. K562 cells were pre-transduced with LVs or IDLVs (as indicated) for 24 hours prior to electroporation with Cas9 plasmid (1ug) and AAV donor template (MOI 2e4). HDR and NHEJ levels were determined by HTS. n=2. Error bars, mean± SD.
