# Supplementary material for: Global and Local Manipulation of DNA Repair Mechanisms to Alter Site-Specific Gene Editing Outcomes in Hematopoietic Stem Cells
Source: Front Genome Ed. 2020 Dec 10;2:601541. doi: 10.3389/fgeed.2020.601541 (PMC8525354; doi:10.3389/fgeed.2020.601541)
Supplement: Supplementary file 1 [file Presentation_1.zip › supp figures correct order/Supplementary Figure 3.PPTX]

## Slide 1
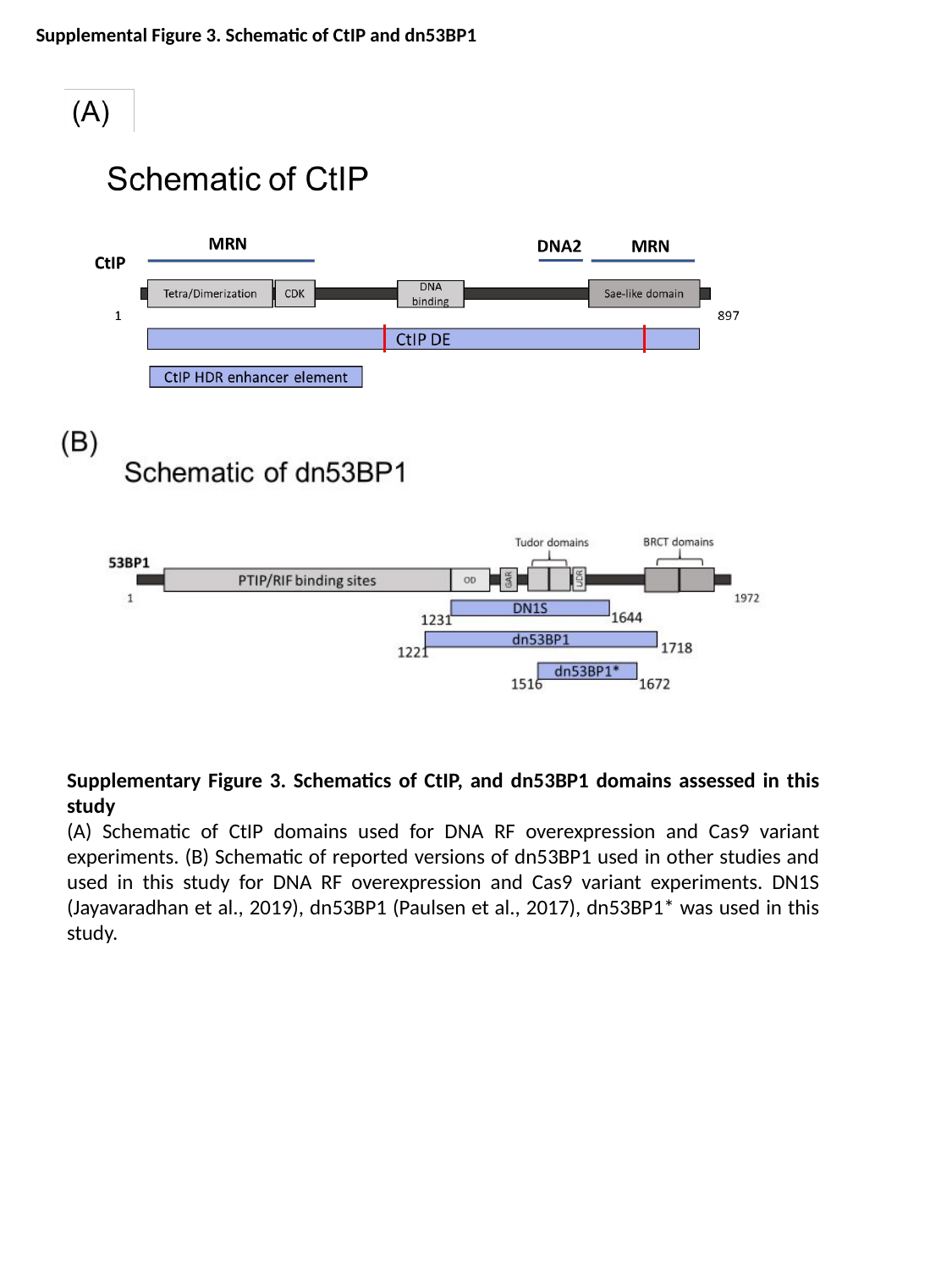

Supplemental Figure 3. Schematic of CtIP and dn53BP1
Supplementary Figure 3. Schematics of CtIP, and dn53BP1 domains assessed in this study
(A) Schematic of CtIP domains used for DNA RF overexpression and Cas9 variant experiments. (B) Schematic of reported versions of dn53BP1 used in other studies and used in this study for DNA RF overexpression and Cas9 variant experiments. DN1S (Jayavaradhan et al., 2019), dn53BP1 (Paulsen et al., 2017), dn53BP1* was used in this study.
