# Supplementary material for: Global and Local Manipulation of DNA Repair Mechanisms to Alter Site-Specific Gene Editing Outcomes in Hematopoietic Stem Cells
Source: Front Genome Ed. 2020 Dec 10;2:601541. doi: 10.3389/fgeed.2020.601541 (PMC8525354; doi:10.3389/fgeed.2020.601541)
Supplement: Supplementary file 1 [file Presentation_1.zip › supp figures correct order/Supplementary Figure 4.PPTX]

## Slide 1
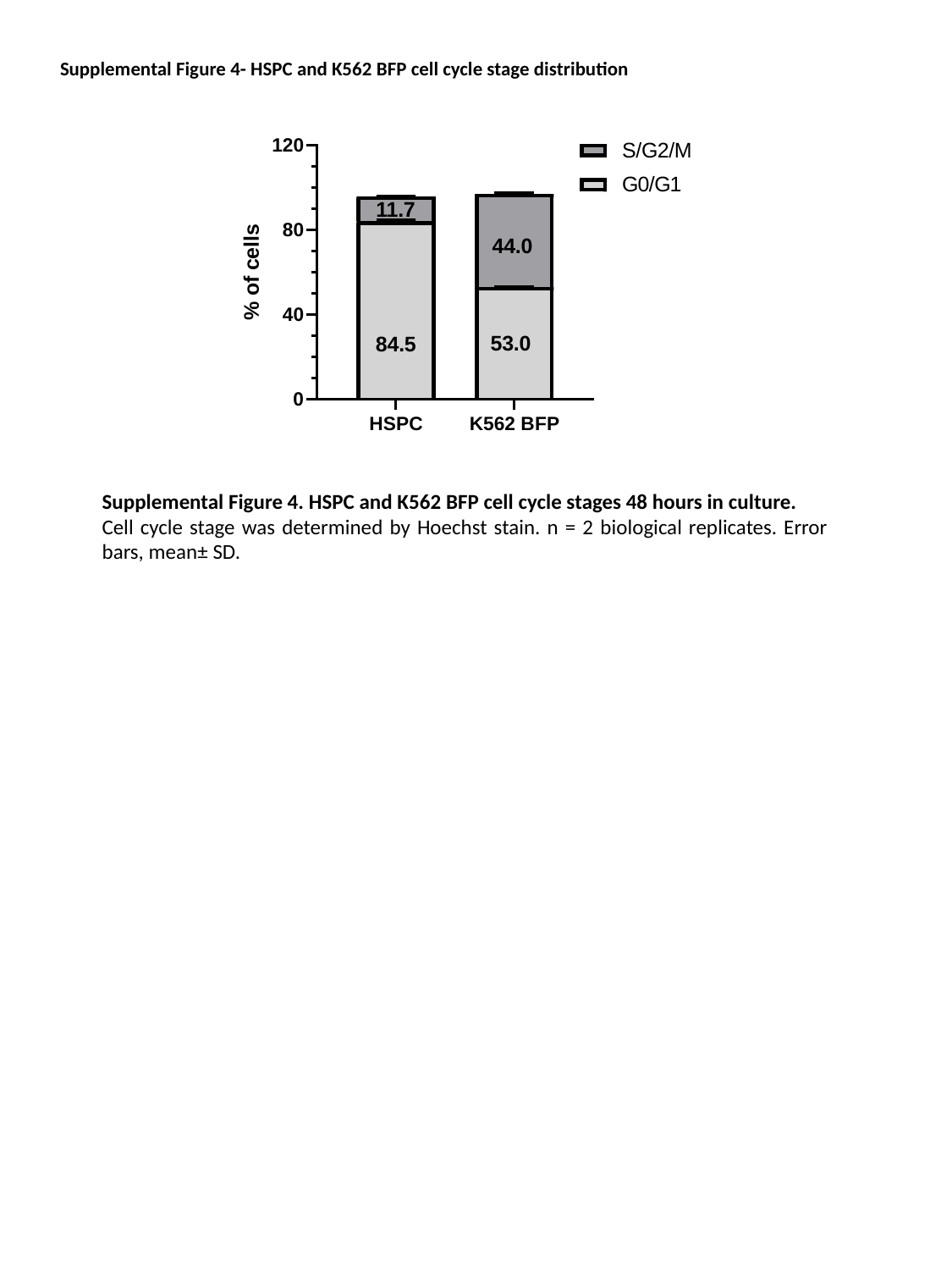

Supplemental Figure 4- HSPC and K562 BFP cell cycle stage distribution
Supplemental Figure 4. HSPC and K562 BFP cell cycle stages 48 hours in culture.
Cell cycle stage was determined by Hoechst stain. n = 2 biological replicates. Error bars, mean± SD.
