# Supplementary material for: Global and Local Manipulation of DNA Repair Mechanisms to Alter Site-Specific Gene Editing Outcomes in Hematopoietic Stem Cells
Source: Front Genome Ed. 2020 Dec 10;2:601541. doi: 10.3389/fgeed.2020.601541 (PMC8525354; doi:10.3389/fgeed.2020.601541)
Supplement: Supplementary file 1 [file Presentation_1.zip › supp figures correct order/Supplementary Figure 5.PPTX]

## Slide 1
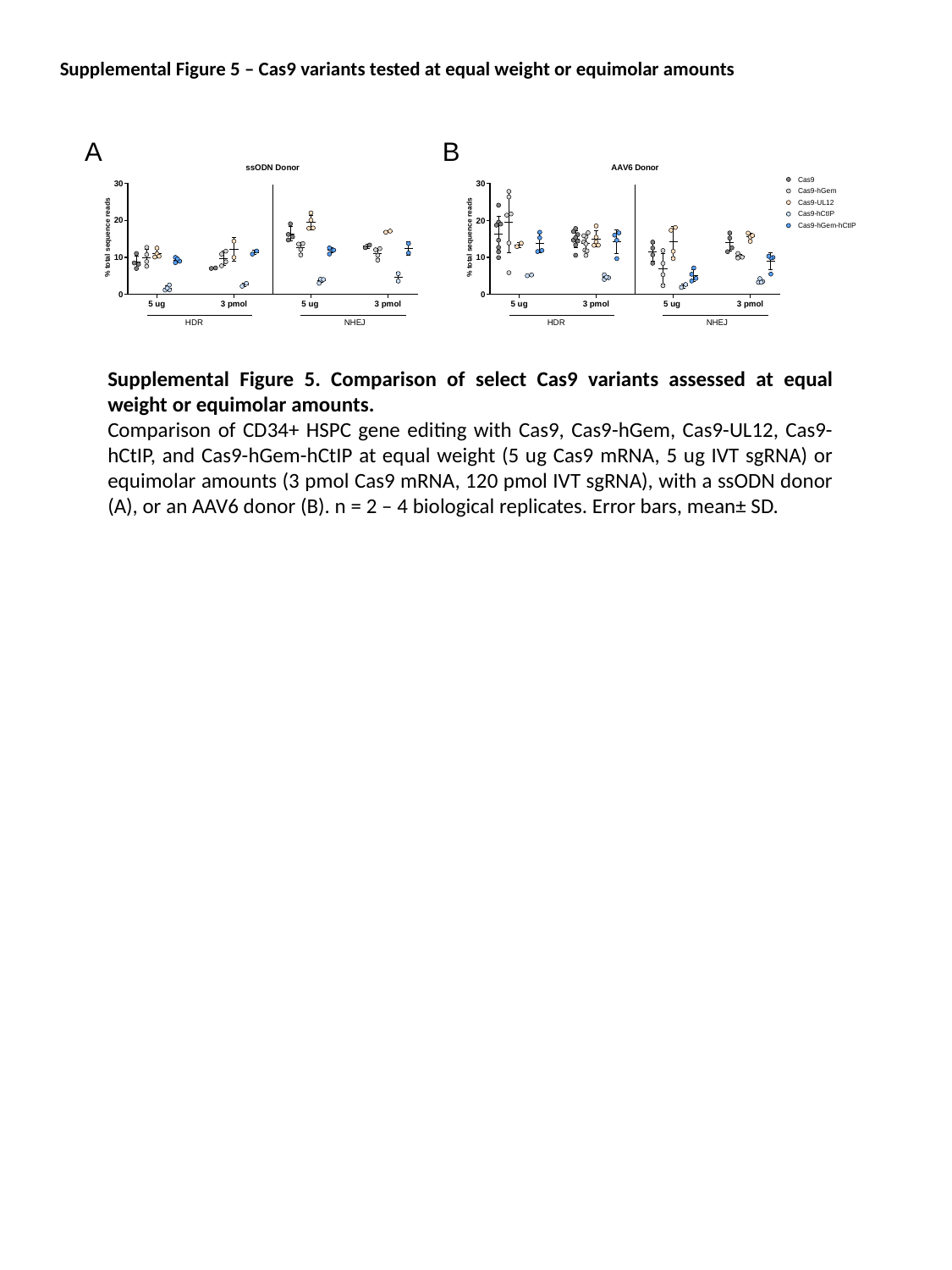

Supplemental Figure 5 – Cas9 variants tested at equal weight or equimolar amounts
Supplemental Figure 5. Comparison of select Cas9 variants assessed at equal weight or equimolar amounts.
Comparison of CD34+ HSPC gene editing with Cas9, Cas9-hGem, Cas9-UL12, Cas9-hCtIP, and Cas9-hGem-hCtIP at equal weight (5 ug Cas9 mRNA, 5 ug IVT sgRNA) or equimolar amounts (3 pmol Cas9 mRNA, 120 pmol IVT sgRNA), with a ssODN donor (A), or an AAV6 donor (B). n = 2 – 4 biological replicates. Error bars, mean± SD.
