# Supplementary material for: Global and Local Manipulation of DNA Repair Mechanisms to Alter Site-Specific Gene Editing Outcomes in Hematopoietic Stem Cells
Source: Front Genome Ed. 2020 Dec 10;2:601541. doi: 10.3389/fgeed.2020.601541 (PMC8525354; doi:10.3389/fgeed.2020.601541)
Supplement: Supplementary file 1 [file Presentation_1.zip › supp figures correct order/Supplementary Figure 6.PDF]

Supplemental Figure 6 – CRISPResso indel spectra of select Cas9 variants

Cas9 + ssODN

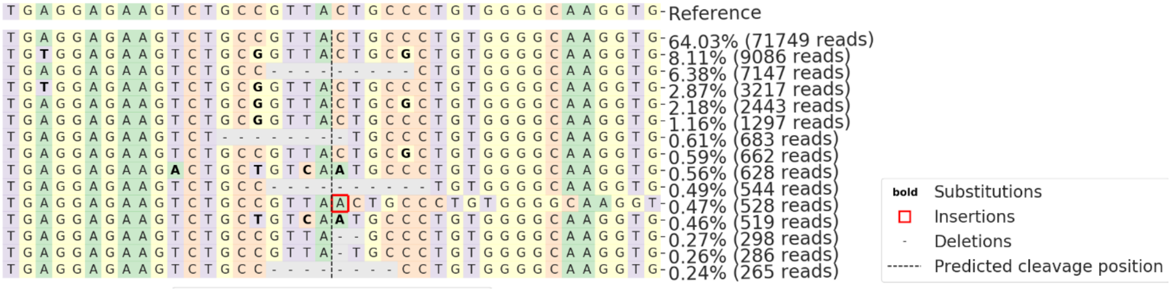

Cas9-hGem + ssODN

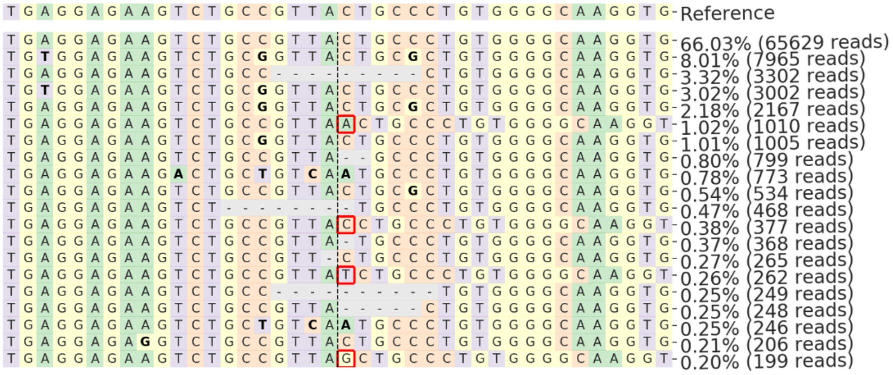

Cas9-hCtIP + ssODN

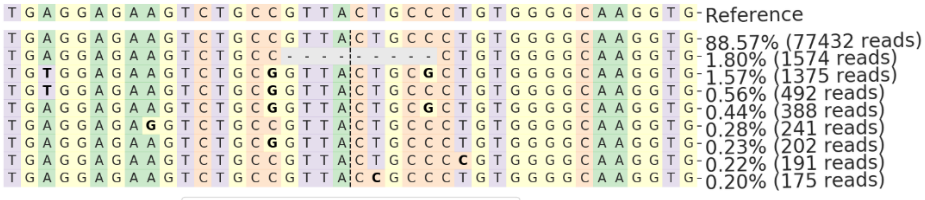

Cas9-hGem-hCtIP + ssODN

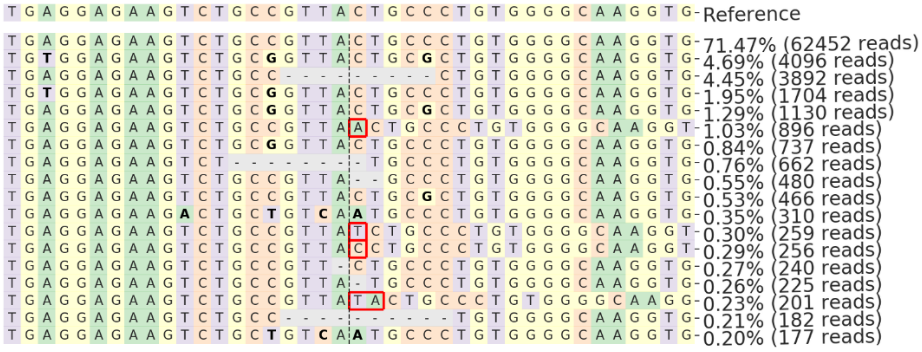

Cas9 + AAV

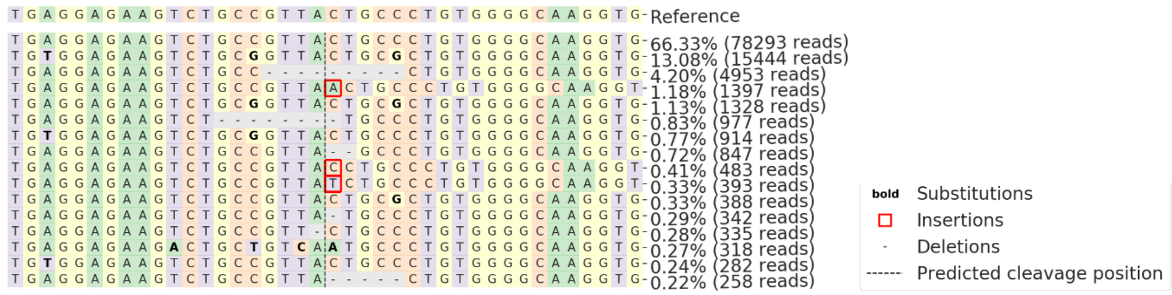

Cas9-hGem + AAV

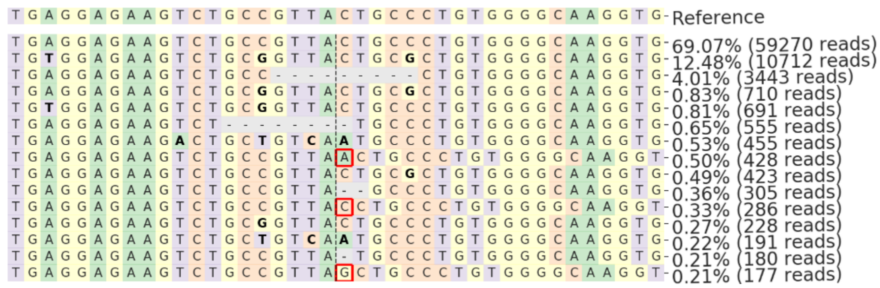

Cas9-hCtIP+ AAV

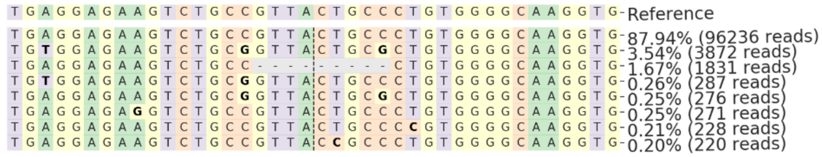

Cas9-hGem-hCtIP + AAV

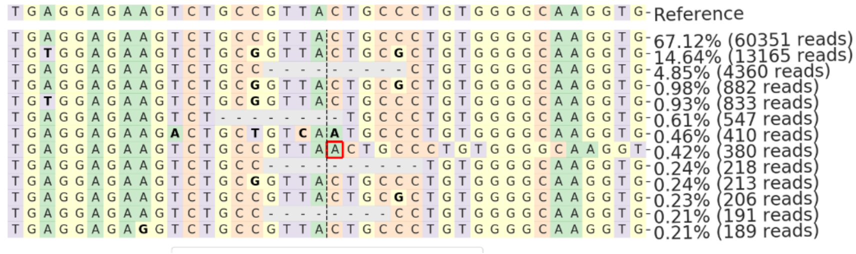

Supplemental Figure 6. Representative indel spectra of select Cas9 variants. Indel spectra from HSPC cells edited with Cas9 variants targeting *HBB* and edited with an ssODN or AAV6 donor.
